# Self-Destroyers

**Published:** 1879-11

**Authors:** 


### SELF-DESTROYERS.

My mind has been painfully impressed, almost daily, with the conviction that the most useful and efficient men in the community are often lost to society, the church, and the world, from a remarkable ignorance of some of the simplest laws of their being. This is not to be wondered at; for, from the nursery, through the primary schools, the academy, the college, the seminary, to the study, not one single lesson is given how to save from a premature death the man who has prepared himself to act in the great drama of the world, by the expenditure of thousands of money, and a score of years of incessant and painful labor, and study, and research.

According to the present generally received views of preparatory professional requirements, the student who has his diploma, the pulpit, or the bar in view, has no time for other than studies which qualify him directly for graduation in the university or the seminary; in fact, so many studies are compressed in such a comparatively short period, that there is not even time for a young gentleman of medium abilities to fully master the most essential elements, or if he does, it is from such close and incessant application that often, with commencement-day, he dies! or if he survives its reaction, his licensure is too frequently clouded and then closed forever, by the stealthy poison of a disease instilled during the diplomatic race. Many a reader of mine will be stricken with the painful remembrance of cases like these in his own sphere of observation, of energies and abilities early blighted, which, had the possessor of them enjoyed the health to work, might have stirred a nation to high resolves.

### WHAT IS NOT DESIGNED.

This Journal is for the people, and is not intended even to admit a single medicinal recipe, although it may be as "*simple*" as syrup of loaf sugar, and as "*harmless*" as a "*vegetable*"—— pill; prussic acid being also "*vegetable.*"

It is not contemplated to issue a series of prose essays on "*Physiology*" or "*Hygiene*," nor to enter into technical and learned disquisitions on the chemical analysis of food, the philosophy of cell life and development, nor indeed any systematic expositions; but simply in short articles, in plain English, to treat on such subjects as may present themselves from time to time, calculated to bear upon the great points,

How to determine disease in its very first approach.

How to arrest it at once by natural agencies.

How to live so as to prevent sickness.

I desire, not promise, that each article shall be complete in itself.

As mine is a consultation practice, and strictly confined to chronic ailments of the throat and lungs, the reader need not be surprised if I give more attention to Bronchitis, Throat Ail, Consumption, and Dyspepsia—because this last is sooner or later inseparably connected with the others—than all others together; in fact they are the main scourges of literary men; but when it is considered that almost all disease comes through the stomach or the lungs, the range will be sufficiently wide for the wildest liberalist.

The design of this Journal is strictly practical; practical in the every-day sense of the word: its teachings will accompany the reader in nearly all the occupations of life; every hour of the day will afford him opportunities of carrying out its principles, not as a weary, fretting task, but in the way of an intelligent and pleasurable observation. The accomplished geologist, while travelling over barren wastes and rocky hills to explore some distant golden district, can make every pebble and every lump of earth minister to his instruction and amusement, without its at all interfering with the main object of his journey: just so may a man's mind be so well stored with intelligent information on the subject of health, and the general laws of life, that observations may be made on these every hour of his waking existence almost, with pleasure and with profit, without at all interfering with the main business of life, and also without having any undesirable influence on mind or body; for he may do this without being forever engaged in looking out for symptoms, aggravating those present or imagining those which are not. In short, the Journal will embrace whatever the Editor thinks will tend to the convenience, comfort, health, and perfection of the physical man, as far as that can be done without the recommendation of any internal medicine, that being the appropriate business of the family physician, and no other than a physician can safely do it. And the man who takes medicine of any kind on his own responsibility, is as sensible as he is said to be who pleads his own cause at the bar, or the wholesale merchant or banker, who, to economize, spends an hour in mending an old shoe, or in sewing on a missing button. A man will seldom attempt to mend his watch that is out of repair, for fear he may do it a greater injury, and yet multitudes of otherwise sensible people are tinkering and tampering with their constitutions by the use or application of remedies of whose qualities they are wholly ignorant, and of whose effects they have never had any experience.

The subjects presented from time to time will be such as—

How to eat.

How to sleep.

How to exercise.

How to dress.

How to walk.

How to read in public.

How to declaim with ease and fluency.

How to select food so as to make it both nutritive and medicinal.

How persons become dyspeptic.

How and why the health of the young so often begins to fail while pursuing an education.

How they may so conduct their studies as to preserve the health, and yet accomplish in the course of the year a larger amount of mental and literary labor.

If an essay is admitted, it will be, among others, from eminent practical dentists, as to the best means of preserving the teeth perfect to the close of life, as perfect teeth are essential to a distinct enunciation, which is of indispensable importance to public men, whether professional or literary.

I will endeavor to make this publication such a one as will be proper for the youth of both sexes to read with increasing interest and attention, so as to induce them early, while the constitution is yet vigorous and unimpaired, so to study the nature and effects, on the human frame, of food, clothing, air, exercise, cheerfulness, system, industry, profitable and interesting employment, as will be an effectual guard against those negligences and indiscretions which so often lay the foundation for an early death

